# Supplementary material for: Physical activity six months after a severe fall – moderating factors in older individuals
Source: BMC Geriatr. 2025 May 29;25:385. doi: 10.1186/s12877-025-06032-2 (PMC12121059; doi:10.1186/s12877-025-06032-2)
Supplement: Supplementary file 2 — Supplementary Material 2 [file 12877_2025_6032_MOESM2_ESM.docx]

Supplementary Material

**Physical Activity Six Months After a Severe Fall – Moderating Factors in Older Individuals**

| Supplementary Tab. 1. | Changes in Sit-to-Stand transfer (STS) and association with moderating factors six months after a fall |
| --- | --- |
| Supplementary Fig. 1. | Study Flow Chart |

Supplementary Tab. 1. Changes in Sit-to-Stand transfer (STS) and association with

moderating factors six months after a fall.

|  | **Estimate** | **95% CI (lower)** | **95% CI (upper)** | **p value** |
| --- | --- | --- | --- | --- |
| Intercept | 49.36 | 45.7 | 52.9 | **6.03×^−88^** |
| Change in STS (T2) | 0.1 | -1.6 | 1.6 | 0.949 |
| Effect of sex (female) | 1.9 | -2.7 | 6.6 | 0.413 |
| Interaction:  Male x fall frequency | -0.5 | -1.6 | 0.7 | 0.422 |
| Interaction:  Female x fall frequency | -0.8 | -1.9 | 0.5 | 0.281 |
| Intercept | 42.2 | 45.8 | 52.5 | **2.62×^−98^** |
| Change in STS (T2) | 0.2 | -1.4 | 1.8 | 0.797 |
| Effect of sex (female) | 0.5 | -3.6 | 4.6 | 0.808 |
| Interaction:  Male x depression | -1.6 | -8.1 | 4.8 | 0.619 |
| Interaction:  Female x depression | 1.4 | -3.5 | 6.3 | 0.569 |
| Intercept | 54.9 | 35 | 74.1 | **<0.01** |
| Change in STS (T2) | 0.1 | -1.5 | 1.7 | 0.898 |
| Effect of sex (female) | -10.9 | -33.9 | 11.9 | 0.347 |
| Interaction:  Male x MoCA | -0.2 | -1.0 | 0.5 | 0.544 |
| Interaction:  Female x MoCA | 0.2 | -0.2 | 0.7 | 0.322 |
| Intercept | 52.7 | 44.4 | 61.1 | **3.94×^−29^** |
| Change in STS (T2) | 0.1 | -1.5 | 1.7 | 0.929 |
| Effect of sex (female) | -2.4 | -12.5 | 7.6 | 0.631 |
| Interaction:  Male x FES-I | -0.3 | -1.2 | 0.4 | 0.390 |
| Interaction:  Female x FES-I | 0.01 | -0.5 | 0.5 | 0.955 |
| Intercept | 61.8 | 37.3 | 86.4 | **<0.01** |
| Change in STS (T2) | 0.1 | -1.5 | 1.7 | 0.947 |
| Effect of sex (female) | -10.4 | -41.5 | 20.7 | 0.511 |
| Interaction:  Male x age | -0.2 | -0.5 | 0.1 | 0.281 |
| Interaction:  Female x age | -0.1 | -0.3 | 0.2 | 0.897 |
| Intercept | 61.3 | 45.5 | 77.1 | **<0.01** |
| Change in STS (T2) | -0.2 | -1.7 | 1.4 | 0.827 |
| Effect of sex (female) | 2.8 | -15.8 | 21.6 | 0.763 |
| Interaction:  Male x BMI | -0.4 | -1.1 | 0.1 | 0.121 |
| Interaction:  Female x BMI | -0.5 | -0.8 | -0.4 | **0.006** |
| Intercept | 49.1 | 45.0 | 53.2 | **1.20×^−71^** |
| Change in STS (T2) | 0.1 | -1.7 | 1.7 | 0.992 |
| Effect of sex (female) | 3.1 | -2.7 | 8.8 | 0.304 |
| Interaction:  Male x unrecovered falls | -0.8 | -7.5 | 5.9 | 0.808 |
| Interaction:  Female x unrecovered falls | -3.8 | -9.1 | 1.6 | 0.156 |
| Intercept | 47.2 | 42.0 | 52.4 | **5.90×^−51^** |
| Change in STS (T2) | -0.5 | -2.1 | 1.1 | 0.530 |
| Effect of sex (female) | 4.7 | -2.4 | 11.8 | 0.194 |
| Interaction:  Male x SPPB | 0.1 | -0.1 | 0.3 | 0.239 |
| Interaction:  Female x SPPB | -0.1 | -0.3 | 0.2 | 0.724 |
| Intercept | 50.1 | 44.9 | 55.1 | **9.42×^−57^** |
| Change in STS (T2) | -0.4 | -1.9 | 1.3 | 0.650 |
| Effect of sex (female) | -1.9 | -8.9 | 5.0 | 0.858 |
| Interaction:  Male x grip strength | -0.1 | -0.2 | 0.1 | 0.537 |
| Interaction:  Female x grip strength | 0.2 | -0.1 | 0.4 | 0.186 |
| Intercept | 55.1 | 45.2 | 64.9 | **4.08×^−24^** |
| Change in STS (T2) | -0.6 | -2.3 | 1.1 | 0.465 |
| Effect of sex (female) | -4.5 | -16.1 | 6.9 | 0.435 |
| Interaction:  Male x gait speed | -7.1 | -17.1 | 2.8 | 0.160 |
| Interaction:  Female x gait speed | 0.1 | -5.5 | 5.7 | 0.974 |

95% CI = 95% confidence interval; BMI, body mass index; FES-I, falls efficacy scale-international^1^;

MoCA, Montreal Cognitive Assessment^2^; SPPB, Short Physical Performance Battery^3^. Results of LMM

are presented; significant values are in bold.

Supplementary Fig. 1.: Study Flow Chart


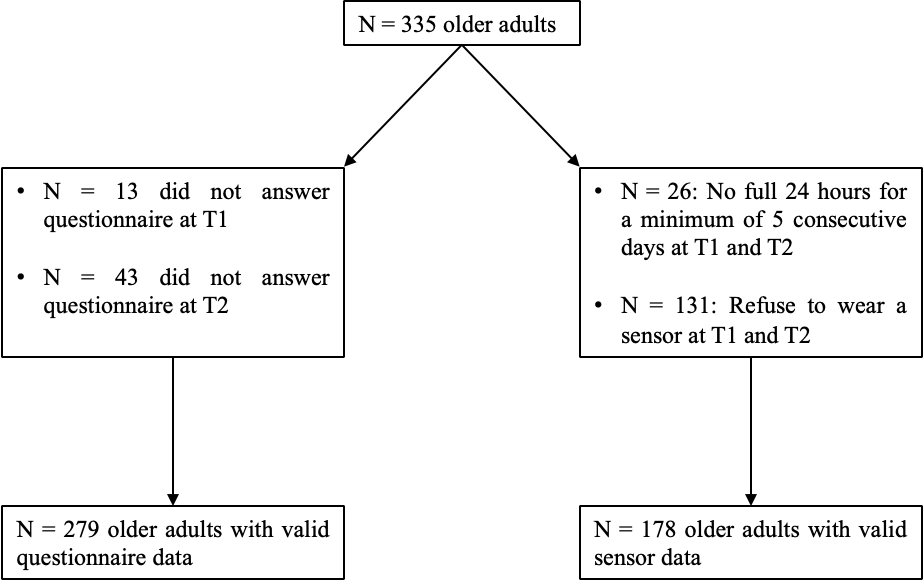


References

1. Kempen GI, Yardley L, Van Haastregt JC, et al. The Short FES-I: a shortened version of the falls efficacy scale-international to assess fear of falling. *Age and ageing*. 2008;37(1):45-50.

2. Dautzenberg G, Lijmer J, Beekman A. Diagnostic accuracy of the Montreal Cognitive Assessment (MoCA) for cognitive screening in old age psychiatry: Determining cutoff scores in clinical practice. Avoiding spectrum bias caused by healthy controls. *International journal of geriatric psychiatry*. 2020;35(3):261-269.

3. Guralnik JM, Simonsick EM, Ferrucci L, et al. A short physical performance battery assessing lower extremity function: association with self-reported disability and prediction of mortality and nursing home admission. *Journal of gerontology*. 1994;49(2):M85-M94.
