# Supplementary material for: Physical activity six months after a severe fall – moderating factors in older individuals
Source: BMC Geriatr. 2025 May 29;25:385. doi: 10.1186/s12877-025-06032-2 (PMC12121059; doi:10.1186/s12877-025-06032-2)
Supplement: Supplementary file 3 — Supplementary Material 3 [file 12877_2025_6032_MOESM3_ESM.docx]

STROBE Statement—checklist of items that should be included in reports of observational studies

|  | Item No. | Recommendation | Page  No. | Relevant text from manuscript |
| --- | --- | --- | --- | --- |
| **Title and abstract** | 1 | (*a*) Indicate the study’s design with a commonly used term in the title or the abstract | 2 | Participants were recruited from the SeFallED study (German Clinical Trials Register ID: 00025949). |
|  |  | (*b*) Provide in the abstract an informative and balanced summary of what was done and what was found | 2 | This study investigates the association between potential moderators and changes in PA in older adults six months after a severe fall. Participants were recruited from the SeFallED study (German Clinical Trials Register ID: 00025949). Moderators were evaluated through a home-based geriatric assessment conducted within four weeks of a severe fall with presentation to the ED. PA was quantified using both sensor-based (n=72 men (75 years), n=106 women (74 years)) and self-reported (n=105 men (74 years), n=174 women (73 years)) assessments. A Linear Mixed Model was used for analysis. Sensor-based PA revealed a significant time effect for step count (p=0.006), indicating an increase in PA six months after a severe fall. Fall history (95% CI: -1,009.5 – (-207.4), p=0.003) and age (95% CI: -315.8 – (-82.5), p<0.01) were significant negative moderators for changes in PA, only in women. No significant changes in self-reported PA were observed after six months (p=0.109). |
| Introduction | | | |  |
| Background/rationale | 2 | Explain the scientific background and rationale for the investigation being reported | 3-4 | Around 40% of community-dwelling older individuals experience a fall once a year, which in 20-30% of the cases requires medical treatment in an emergency department (ED) (1, 2). Falls often cause concerns about falling, impair activities of daily living, and reduce cognitive function and physical activity (3, 4).  Physical activity (PA) is defined as any bodily movement produced by skeletal muscles that demand energy expenditure and is often categorized into occupational, sports, conditioning, household or other activities (5-8). Changes in PA following a fall often indicate the beginning of a downward spiral. Fall-related consequences, such as increased concerns about falling, may lead to activity avoidance. While this avoidance may reduce immediate risk exposure, it can have detrimental long-term effects by contributing to social isolation and the progressive decline of physical functioning (9, 10). Monitoring PA offers valuable insights into early behavioral adaptations that may precede measurable declines in physical function such as balance and gait impairments. Especially in vulnerable populations, PA can serve as a proxy for early, modifiable changes that contribute to long-term functional decline (8). Therefore, early identification of longitudinal changes in PA may help to appropriately target individuals at highest risk for further functional decline and recurrent falls.  PA in older adults with a history of falls has been investigated in previous literature cross-sectionally, with most of the studies using self-reproted measures e.g., validated questionnaires (5, 6, 11). Self-reported measures of PA underlie response bias as well as recall bias which can affect data accuracy, and data collection is often challenging in vulnerable populations such as individuals with cognitive impairment. Contrary to validated PA-questionnaires wearable motion sensors provide an objective and more reliable understanding of PA in older adults (12, 13). A recent systematic review provided a deeper insight into the usage of accelerometers to measure PA in community-dwelling older adults (14). The authors identified seven studies, which assessed PA by using the ActivPal ^TM^ as a wearable motion sensor. The most common parameters used in these studies were step count, time spent walking, sit-to-stand transition, and sedentary time (14).  One of the longitudinal studies that utilized a wearable motion sensor to observe individuals with a history of falls, reported significant differences in PA after a three-year follow-up period, attributed to the initial fall event. Individuals who experienced an injurious fall reduced their average daily step count and daily activity minutes significantly more than those who did not experience an injurious fall over a three-year follow-up period (15). Another study examined the moderating factors of various PA components related to falls and concerns about falling in older adults, as well as whether participation in a physical exercise program could reduce these risks (16). The study revealed a significant association between falls and one-leg standing time, long-term PA participation, fall history and concerns about falling. Additionally, concerns about falling were linked to age, sex, and different PA components (16). However, evidence remains limited regarding the moderating factors that influence changes in PA following a fall, particularly in high-risk individuals. Previous studies have primarily focused on community-dwelling populations (9, 10), older adults recovering from injuries such as hip fractures (17), or those with glaucoma (15, 18). According to global guidelines for falls prevention, individuals presenting to the ED after a fall are classified as a high risk group (8). However, this population is heterogenous, and not all individuals follow the same post-fall trajectory. Identifying red flags – such as behavioral adaptations that precede measurable functional decline – can help refine risk stratification and guide tailored treatment approaches (19, 20). |
| Objectives | 3 | State specific objectives, including any prespecified hypotheses | 4 | Therefore, the aim of this study was to investigate whether clinically relevant moderators such as physical function, cognition, depressive symptoms, fall-related factors, and personal factors were associated with changes in sensor-based and self-reported PA, in older men and women six months after a severe fall with presentation to the ED. |
| Methods | | | |  |
| Study design | 4 | Present key elements of study design early in the paper | 5 | Participants were recruited from the SeFallED (“Sentinel Fall Presenting to the Emergency Department”) study, which is currently ongoing at the Carl von Ossietzky University Oldenburg, Germany (21). Complete baseline data within four weeks after a severe fall (T1) and after six months (T2) from the observational, prospective part of the study were used. Participants enrolled between November 2021 and December 2023 were included in this sub-study. |
| Setting | 5 | Describe the setting, locations, and relevant dates, including periods of recruitment, exposure, follow-up, and data collection | 5-6 | Participants had to meet the following inclusion criteria: 1) age 60 years or above, 2) presented to the ED of the Klinikum Oldenburg or Evangelisches Krankenhaus Oldenburg after a fall and were discharged within 72 h, 3) informed consent. Exclusion criteria are as follows: 1) life expectancy of less than 3 months, 2) unstable medical, neurological or psychiatric condition, 3) bedridden or being unable to walk without support of another person, 4) residence more than 40 km away from the research center, 5) acute psychosis or social aggression, 6) inability to communicate verbally in German or English (21).  Study overview  Subjects gave their written informed consent for further contact in the ED to a study nurse, who approached individuals meeting the inclusion criteria of the study. After being contacted via telephone, a trained member of the study team conducted a home visit to obtain informed consent for participation. The whole recruitment procedure has been described comprehensively in a previous article (19). During the first home visit (T1), an extensive structured and standardized geriatric assessment was conducted. A list of the assessment battery has been published before, but included information about the participants, fall history, concerns about falling, depressive symptoms, activities of daily living, and functional performance (21). Due to individual preferences, not all participants consented to wear the sensor used for measuring PA, and in a few cases, questionnaire data were incomplete. Sensor use was an additional source of data and required separate consent; participants who declined remained eligible for other study procedures. Consequently, sample sizes varied slightly across data sources, and a baseline comparison was performed to assess group comparability. The inclusion criteria were intentionally broad to capture a heterogeneous sample reflective of older adults typically presenting to the ED after a fall. This approach included individuals with cognitive impairment. In such cases, caregivers or family members supported data collection efforts, particularly for questionnaire-based assessment. The second home visit after 6 months (T2) was conducted similarly to the first home visit (T1). |
| Participants | 6 | (*a*) *Cohort study*—Give the eligibility criteria, and the sources and methods of selection of participants. Describe methods of follow-up  *Case-control study*—Give the eligibility criteria, and the sources and methods of case ascertainment and control selection. Give the rationale for the choice of cases and controls  *Cross-sectional study*—Give the eligibility criteria, and the sources and methods of selection of participants | 5-6 | Participants had to meet the following inclusion criteria: 1) age 60 years or above, 2) presented to the ED of the Klinikum Oldenburg or Evangelisches Krankenhaus Oldenburg after a fall and were discharged within 72 h, 3) informed consent. Exclusion criteria are as follows: 1) life expectancy of less than 3 months, 2) unstable medical, neurological or psychiatric condition, 3) bedridden or being unable to walk without support of another person, 4) residence more than 40 km away from the research center, 5) acute psychosis or social aggression, 6) inability to communicate verbally in German or English (21).  Study overview  Subjects gave their written informed consent for further contact in the ED to a study nurse, who approached individuals meeting the inclusion criteria of the study. After being contacted via telephone, a trained member of the study team conducted a home visit to obtain informed consent for participation. The whole recruitment procedure has been described comprehensively in a previous article (19). During the first home visit (T1), an extensive structured and standardized geriatric assessment was conducted. A list of the assessment battery has been published before, but included information about the participants, fall history, concerns about falling, depressive symptoms, activities of daily living, and functional performance (21). Due to individual preferences, not all participants consented to wear the sensor used for measuring PA, and in a few cases, questionnaire data were incomplete. Sensor use was an additional source of data and required separate consent; participants who declined remained eligible for other study procedures. Consequently, sample sizes varied slightly across data sources, and a baseline comparison was performed to assess group comparability. The inclusion criteria were intentionally broad to capture a heterogeneous sample reflective of older adults typically presenting to the ED after a fall. This approach included individuals with cognitive impairment. In such cases, caregivers or family members supported data collection efforts, particularly for questionnaire-based assessment. The second home visit after 6 months (T2) was conducted similarly to the first home visit (T1). |
|  |  | (*b*) *Cohort study*—For matched studies, give matching criteria and number of exposed and unexposed  *Case-control study*—For matched studies, give matching criteria and the number of controls per case |  |  |
| Variables | 7 | Clearly define all outcomes, exposures, predictors, potential confounders, and effect modifiers. Give diagnostic criteria, if applicable | 6-9 | Outcome measures  Physical Activity  Sensor-based PA was measured using a three-axial accelerometer (activPAL4^TM^, PAL Technologies Ltd., Glasgow, UK) (13), which was attached to the participants’ right thigh by a trained member of the study team as part of the comprehensive assessment during both home visits (T1 and T2). An additional consent form was used concerning the collection of sensor data. If participants provided their consent, they were instructed to wear the sensor for seven consecutive days. A minimum of 24 hours of data collection for at least five consecutive days was required for inclusion of the data in the analysis, as recommended by previous literature (22). Parameters derived from the wearable device have been validated for measuring PA in community-dwelling older individuals (13). Accordingly, we analyzed number of steps per day, time spent walking (minutes), sedentary time (minutes) and the number of sit-to-stand transitions (14).  Additionally, self-reported PA was assessed using the German-PAQ-50+ (23) during both home visits (T1 and T2). The German-PAQ-50+ (23) is a well established questionnaire in individuals over the age of 50 years and a feasible measure of self-reported PA (24). We used PAQ-50+-total scores to assess the total time spent on PA (minutes) for further analysis.  Moderators  Moderators were assessed as part of the comprehensive geriatric assessment at baseline (T1).  *Cognitive function*  Montreal Cognitive Assessment (MoCA) was used to assess cognitive function, which is a valid screening tool for global cognition with higher scores indicating better cognitive function (25). The total score was used as a moderator.  *Depressive Symptoms*  Depressive symptoms were either assessed by total score of the Depression in Old Age Scale (DIA-S) (26), ranging from 0 (no depressive symptoms) to 10 points (maximal amount of depressive symptoms) with cut-off for probable depressive symptoms at 3 points, or Cornell Depression Scale (27), ranging from 0-38 points with a score >10 points indicating major depressive symptoms. The Cornell Depression Scale was used instead of the DIA-S, when participants had severe cognitive impairment (MoCA <18 points). The Cornell Depression Scale was administered with the assistance of participants’ family members or caregivers. For subsequent statistical analysis, a new variable, “presence of depressive symptoms” (yes/no) was created based on the participants’ total scores from either the DIA-S or the Cornell Depression Scale.  *Physical function*  The Short Physical Performance Battery (SPPB) is a commonly used assessment to investigate lower extremity functioning in older adults (28). The assessment consists of a parallel, semi tandem and a tandem stance for ten seconds to assess standing balance performance, a 4-meter walking test conducted at participants’ usual pace to assess gait speed and a five times chair-stand test to assess the ability to get up as fast as possible from a chair without support of the arms. Adding the sub-scores of standing balance, walking, as well as chair-stand components of the SPPB (ranging from 0-4), builds the SPPB total score (ranging from 0-12), with lower scores indicating worse functioning (28). Due to insufficient space at participants’ homes, a modified version with a three meter walk instead of four meters was conducted, which is in line with previous research (29, 30). Participants’ total score of lower extremity functioning and gait speed were included as moderators.  Grip strength, which is a proxy for muscle strength, was assessed using a Jamar^®^ hand dynamometer, which has proven a good test-retest reliability, and inter-, as well as intra-rater reliability. Participants were instructed to sit on a chair with the elbow in 90° flexion, shoulder adducted and without support surface of the armrest, as described in the American Society of Hand Therapists (ASHT) grip-strength protocol (31). Participants were instructed to squeeze the dynamometer as hard as possible for three alternating times. Mean grip score in kilograms from the three trials of the dominant hand were used for further analysis.  *Fall-related factors*  Fall history was assessed by asking participants, “how many falls have you experienced within the last 12 months?”. Therefore, fall history reflects the total number of falls in the last 12 months before attending the ED, while the initial fall leading to study inclusion was not included in that variable.  Concerns about falling were quantified by using the Short falls efficacy sale-international (FES-I) (32), which is a reliable and clinically practical measure. FES-I consists of 7 different items describing the presence of concerns about falling during daily activities for example while getting dressed/undressed or while getting in/out of a chair. Participants were advised to choose, whether they are not at all (1), somewhat (2), fairly (3), or very concerned (4) to fall during daily activities. Total score of FES-I with maximum score of 28 points indicating severe concerns about falling, were used for interpretation.  In addition to fall history and concerns about falling, “unrecovered” and “recovered” falls were determined by asking participants, if they were able to get up unaided (= recovered) after their severe fall leading to study inclusion, or whether they were unable to get up on their own (= unrecovered).  *Personal factors*  Age in years and body mass index, calculated from body mass in kilograms and body height in centimeters as part of the comprehensive geriatric assessment, were included into the statistical model. |
| Data sources/ measurement | 8* | For each variable of interest, give sources of data and details of methods of assessment (measurement). Describe comparability of assessment methods if there is more than one group | Not applicable | Not applicable |
| Bias | 9 | Describe any efforts to address potential sources of bias | 6 | The inclusion criteria were intentionally broad to capture a heterogeneous sample reflective of older adults typically presenting to the ED after a fall. This approach included individuals with cognitive impairment. In such cases, caregivers or family members supported data collection efforts, particularly for questionnaire-based assessment. |
| Study size | 10 | Explain how the study size was arrived at | 10 | No a priori power calculation was performed, as this study uses a sub-sample from the larger prospective study and analyses performed were exploratory. |

Continued on next page

| Quantitative variables | 11 | Explain how quantitative variables were handled in the analyses. If applicable, describe which groupings were chosen and why | 9 | Participants were included in our statistical analyses if they had valid accelerometer data and completed questionnaires at both baseline (T1) and six months later (T2), as well as valid data for at least one of the predefined moderators at baseline (T1). Statistical analysis were conducted separately for participants with valid accelerometer datasets and those with only valid questionnaire data sets. Complete case analyses were used.  To identify the association between changes in sensor-based and self-reported PA, we conducted a Linear Mixed Model (LMM) analysis with sex (male and female) as a fixed between-subjects effect. |
| --- | --- | --- | --- | --- |
| Statistical methods | 12 | (*a*) Describe all statistical methods, including those used to control for confounding | 9 | To identify the association between changes in sensor-based and self-reported PA, we conducted a Linear Mixed Model (LMM) analysis with sex (male and female) as a fixed between-subjects effect. Additionally, baseline cognitive function, depressive symptoms, physical function, fall-related factors, and personal factors were included as moderating factors. Age (in years) and number of previous falls before initial fall event leading to study inclusion (number of falls in the last 12 months) were dichotomized using the following cut-off values: <63 years ≥ vs. 63 years and <1 fall vs. ≥1 fall.  For the analyses, we used SPPS for MacOS (version 28.0; SPSS Inc., Chicago, IL, USA) and R ((R Core Team, 2023 Version 4.3.2 (2023-10-31)) with the ‘nlme’ package for LMM analyses with longitudinal measurements.  Inferential statistics are intended to be exploratory (hypothesis-generating) rather than confirmatory, and should be interpreted accordingly. No a priori power calculation was performed, as this study uses a sub-sample from the larger prospective study and analyses performed were exploratory. The local significance level is set at 0.05, and no adjustment for multiple testing is performed. |
|  |  | (*b*) Describe any methods used to examine subgroups and interactions | 9 | To identify the association between changes in sensor-based and self-reported PA, we conducted a Linear Mixed Model (LMM) analysis with sex (male and female) as a fixed between-subjects effect. Additionally, baseline cognitive function, depressive symptoms, physical function, fall-related factors, and personal factors were included as moderating factors. Age (in years) and number of previous falls before initial fall event leading to study inclusion (number of falls in the last 12 months) were dichotomized using the following cut-off values: <63 years ≥ vs. 63 years and <1 fall vs. ≥1 fall. |
|  |  | (*c*) Explain how missing data were addressed | 9 | Participants were included in our statistical analyses if they had valid accelerometer data and completed questionnaires at both baseline (T1) and six months later (T2), as well as valid data for at least one of the predefined moderators at baseline (T1). Complete case analyses were used. |
|  |  | (*d*) *Cohort study*—If applicable, explain how loss to follow-up was addressed  *Case-control study*—If applicable, explain how matching of cases and controls was addressed  *Cross-sectional study*—If applicable, describe analytical methods taking account of sampling strategy | Not applicable | Not applicable |
|  |  | (*e*) Describe any sensitivity analyses | Not applicable | Not applicable |
| Results | | | | |
| Participants | 13* | (a) Report numbers of individuals at each stage of study—eg numbers potentially eligible, examined for eligibility, confirmed eligible, included in the study, completing follow-up, and analysed | 10 | Out of 335 participants at baseline, 279 older adults (n=105 male and n=174 female participants) completed the PAQ-50+ at both time points. In total, 178 older adults (n=72 male and n=106 female participants) had complete accelerometer data for both time points. While n=131 participants refused to wear an accelerometer either at T1 or T2, n=26 data sets did not incorporate a full 24-hour cycle for 5 five consecutive days either at T1 or T2 (see supplementary fig. 1). |
|  |  | (b) Give reasons for non-participation at each stage | 10 | Out of 335 participants at baseline, 279 older adults (n=105 male and n=174 female participants) completed the PAQ-50+ at both time points. In total, 178 older adults (n=72 male and n=106 female participants) had complete accelerometer data for both time points. While n=131 participants refused to wear an accelerometer either at T1 or T2, n=26 data sets did not incorporate a full 24-hour cycle for 5 five consecutive days either at T1 or T2 (see supplementary fig. 1). |
|  |  | (c) Consider use of a flow diagram | Supplements | (see supplementary fig. 1). |
| Descriptive data | 14* | (a) Give characteristics of study participants (eg demographic, clinical, social) and information on exposures and potential confounders | 10 | Within the PAQ-50+ group, women reported higher concerns about falling at baseline in comparison to men (p=0.035). Cognitive function was significantly lower for men compared to women within the PAQ-50+ group (p=0.015), as well as within the ActivPal group (p=0.031), while between-group differences indicated lower cognitive function for men in the PAQ-50+ group than women in the ActivPal-group (p=0.007). Hand grip strength differed between men and women in both groups (p<0.001) (tab. 1.) No further differences were identified for age, body mass index, education, fall history and diseases, activities of daily living and functional performance (tab. 1.). |
|  |  | (b) Indicate number of participants with missing data for each variable of interest | 10 | Out of 335 participants at baseline, 279 older adults (n=105 male and n=174 female participants) completed the PAQ-50+ at both time points. In total, 178 older adults (n=72 male and n=106 female participants) had complete accelerometer data for both time points. While n=131 participants refused to wear an accelerometer either at T1 or T2, n=26 data sets did not incorporate a full 24-hour cycle for 5 five consecutive days either at T1 or T2 (see supplementary fig. 1). |
|  |  | (c) *Cohort study*—Summarise follow-up time (eg, average and total amount) | Not applicable | Not applicable |
| Outcome data | 15* | *Cohort study*—Report numbers of outcome events or summary measures over time | Not applicable | Not applicable |
|  |  | *Case-control study—*Report numbers in each exposure category, or summary measures of exposure | Not applicable | Not applicable |
|  |  | *Cross-sectional study—*Report numbers of outcome events or summary measures | Not applicable | Not applicable |
| Main results | 16 | (*a*) Give unadjusted estimates and, if applicable, confounder-adjusted estimates and their precision (eg, 95% confidence interval). Make clear which confounders were adjusted for and why they were included | 10-11 | *Main ouctomes – LMM for sensor-based PA*  The linear mixed model for sensor-based PA revealed a significant time effect for step count (p=0.006) and stepping time (p=0.031), indicating an increase in PA six months after a severe fall (T2). However, sit-to-stand transitions (p=0.949) and sedentary time (p=0.163) did not change.  *LMM - Step count*  Notably, women significantly increased the average number of steps per day by +1,777.9 (95% CI: 292.2 – 3,263.7, p=0.019) compared to male participants (tab. 2). Regarding moderating factors, fall history (95% CI: -1,009.5 – (-207.4), p=0.003), age (95% CI: -315.8 – (-82.5), p<0.01), and unrecovered falls (95% CI: -4,281.5 – (-1,027.0), p=0.003) were associated with a decrease in average number of steps, but this effect was observed only in female participants (tab. 2).  *LMM – Stepping time*  Fall history (95% CI: -9.4 – (-1.6), p=0.005), age (95% CI: -2.6 – (-1.2), p<0.01), BMI ((95% CI: -2.8 – (-0.5), p=0.003), and unrecovered falls (95% CI: -37.7– (-8.8), p=0.001), were associated with a reduction in stepping time, whereas grip strength (95% CI: 0.4 – 2.0, p=0.002) was associated with an increase in stepping time, specifically in female participants. Moreover, concerns about falling were negatively associated with stepping time for both male (95% CI: -6.0– (-0.9), p=0.007) and female individuals (95% CI: -4.5– (-1.2), p<0.01; tab. 4.).  *LMM – Sedentary time*  For sedentary time, we could identify a positive association with fall history (95% CI :1.0 – 10.2, p=0.016), concerns about falling (95% CI: 2.5 – 25.9, p=0.017), age (95% CI: 0.1 – 4.6, p=0.037), BMI (95% CI: 2.7– 9.1, p<0.01), and unrecovered falls in female (95% CI: 12.4 – 100.7, p=0.012), but not in male individuals (tab. 3.) Additionally, female participants exhibited a decrease in sedentary time by -54.1 minutes at T2 in comparison to male participants (95% CI: -96.8 – (-11.43), p=0.013).  *LMM – Sit to stand transfer*  A negative association between number of sit to stand transfer was found for BMI (95% CI: -0.8 – (-0.4), p=0.006), but only for women (supplementary tab. 1.).  *Main ouctomes – LMM for self-reported PA*  In contrast to sensor-based PA, we did not observe any changes in self-reported PA in older adults six months after a severe fall (p=0.109). For female participants, fall history (95% CI: -13.9 – (-0.1), p=0.047) was negatively associated with self-reported PA, while physical function showed a positive association (95% CI: 0.4 – 3.5, p=0.01). Concerns about falling (M: 95% CI: -11.2 – (-4.6), p<0.01; W: 95% CI: -1.7 – (-1.7), p<0.01), age (M: 95% CI: -4.6 – (-1.6), p<0.01; W: 95% CI: -5.0 – (-2.7, p<0.01), and unrecovered falls (M: 95% CI: -78.2 – (-19.0, p=0.001; W: 95% CI: -48.2 – (-3.5), p=0.023) were negatively associated. While cognition (M: 95% CI: 1.9 – 9.9, p=0.003; W: 95% CI: 2.2 – 7.6, p<0.01), and grip strength (M: 95% CI: 1.2 – 2.9, p<0.01; W: 95% CI: 0.3 – 2.9, p=0.01) were positively associated with self-reported PA in both, men and women (tab 5.). |
|  |  | (*b*) Report category boundaries when continuous variables were categorized | Not applicable | Not applicable |
|  |  | (*c*) If relevant, consider translating estimates of relative risk into absolute risk for a meaningful time period | Not applicable | Not applicable |

Continued on next page

| Other analyses | 17 | Report other analyses done—eg analyses of subgroups and interactions, and sensitivity analyses | 12-13 | *LMM for Cut-off values – sensor-based PA*  The analysis identified critical cut-off values for sensor-based PA. Female sex combined with an age of 70 years or older was associated with a significant increase in sedentary time by +43.5 minutes per day (95% CI: 0.5 – 86.6, p=0.04). Additionally, female sex and an age of 66 years or older were linked to a significant decrease in the number of steps per day by -2,205.7 (95% CI: -4,062.6 – (-348.8), p=0.02).  Female sex and a fall history of at least one fall significantly reduced stepping time by -18.7 minutes per day (95% CI: -33.5 – (-3.9), p=0.013), and number of steps per day by -1,847.8 (95% CI: -3,341.7 – (-348.1), p=0.015). Moreover, female sex and a fall history of at least one fall significantly increased sedentary time by +47.1 minutes per day (95% CI: 4.4 – 89.8, p=0.0310).  *LMM for Cut-off values – self-reported PA*  An age of 61 years and above, combined with sex, significantly reduced total time spent on PA by -128.4 minutes in male participants (95% CI: -216.1 – (-40.8), p=0.004) and by -89.4 minutes in female participants (95% CI: -158.0 – (-20.7), p=0.01). |
| --- | --- | --- | --- | --- |
| Discussion | | | | |
| Key results | 18 | Summarise key results with reference to study objectives | 13-17 | **Discussion**  Older adults increased their sensor-based PA by 545.8 steps per day and 6.2 minutes of stepping time six months after a severe fall, whereas self-reported PA remained unchanged. In female participants, moderating factors such as one or more falls within 12 months preceding the severe fall requiring ED care, advanced age (66 years and older), and unrecovered falls were associated with decreased sensor-based PA by -2,205.7 steps per day over time following the severe fall. Irrespective of sex, cognitive and physical function were positively associated with changes in self-reported PA, whereas concerns about falling and unrecovered falls negatively impacted it over time.  *Sensor-based PA*  In our study, participants increased their PA six months after a severe fall based on accelerometer data. These results contrast with findings by Jian-Yu and colleagues, who reported that individuals who experienced an injurious fall, decreased their PA after one year (15). Notably, the study by Jian-Yu et al. included pre-fall PA data, enabling within-subject comparisons that are not possible in our analysis. This fundamental design difference limits the direct comparability of our findings. Moreover, their study population – individuals with glaucoma – represents a more specific subgroup at high-risk, whereas our cohort reflects a broader, more heterogeneous population of older adults presenting to the ED after a severe fall. Despite these differences, both studies underscore the importance of personalized interventions, as both pre-existing conditions and the fall event itself relevantly shape subsequent PA trajectories (8).  The discrepancy between the studies may also arise from differences in methodological approaches. Our study assessed participants within four weeks after a severe fall requiring ED presentation, whereas Jian-Yu and colleagues evaluated participants with Glaucoma once a year over the course of four years. The immediate assessment of PA in our study does not represent a true baseline measurement, as PA might be acutely reduced due to fall-related injuries and the recent severe fall itself (33). Consequently, the observed increase in sensor-based PA six months after a fall may reflect either a recovery from an initial, short-term decline in activity following the fall that prompted inclusion in the SeFallED study, or the possibility that some participants resumed activity immediately, despite experiencing fall-related consequences such as pain or concerns about falling. A decrease in PA during the first six months after a severe fall could be particularly concerning, as it may signal the onset of future functional decline. Therefore, the early identification of longitudinal changes in PA—along with key moderating risk factors—is crucial for preventing social isolation and activity avoidance in older adults at high risk of falling. While a reduction in PA may, in some cases, reflect a behavioral adaptation to increased perceived or actual fall risk, it may also signify the onset of social withdrawal and a decline in functional independence. This dual interpretation highlights the complexity of post-fall behavioral responses, particularly considering previous findings suggesting a U-shaped association between PA levels and fall risk exposure[Chantanachai, 2024 #222](34). To address this, tailored interventions—such as supervised exercise or the use of mobility aids—can support safe and sustained engagement in physical activity.  Our results extend previous findings by shifting from a cross-sectional to a longitudinal perspective on moderating factors of PA in older adults following a severe fall. However, future studies may benefit from repeated or extended monitoring periods to capture day-to-day fluctuations and provide more stable estimates of PA (35, 36). While continuous monitoring using everyday wearable devices – such as smartphones, smartwatches or fitness trackers – is becoming increasingly feasible (37), these technologies currently lack the precision and clinical validity of research-grade tools. Therefore, their integration into clinical practice should be approached carefully.  *Self-reported PA*  While sensor-based PA increased, self-reported PA did not change for either male or female participants in our study. This discrepancy could be due to participants either not perceiving any impairments in PA, leading to an overestimation of PA at baseline, or undererstimating their PA at follow-up. We speculate that overestimation at baseline is more likely, as older adults aged 65-84 years often overestimate their PA compared to sensor-based measured data. Previous studies have attributed this overestimation to social desirability response bias, where participants may exaggerate their PA due to the known benefits of PA and exercise (38, 39). Furthermore, PA measured by a questionnaire represents a subjective estimation in a typical week. Therefore accuracy cannot be as high as data given by a sensor-based solution.  *Moderating factors and sex-specific variations*  In addition to consistent findings on advanced age (40), with 66 year identified as a critical cut-off in our sensor-based PA data, history of falls also emerged as a significant moderator. Given that a history of multiple falls has been identified as a significant risk factor for functional decline in the latest version of the global guidelines for falls prevention and management for older adults (8), it is important not only to confirm the initial fall that led to the ED visit, but also to ask about previous falls within the past year. This information, which is often overlooked in acute care settings, provides critical insight into a patient’s broader risk profile. Including a brief question about recent fall history is both time-efficient and easy to administer, and can help clinicians identify individuals who may benefit from targeted follow-up treatment (41, 42).  In our study, moderating factors in men were only associated with self-reported PA, while in women, they influenced both, self-reported and sensor-based PA. A previous study, which analyzed sex differences in PA, found that male participants, who experienced frequent falls significantly reduced their leisure activites (e.g., biking), and both light and heavy household chores (e.g., sweeping, washing windows) over time compared to those who fell less frequently or not at all. In contrast, women maintained similar levels of leisure activity and household work, showing no decline in PA. The authors proposed that women’s engagement in these activities was not significantly affected by falls. Therefore, further differences between the PA assessments used in our study, may be explained by methodogical variations.  *Differences between sensor- and questionnaire-based PA*  The ActivPal, used for sensor-based PA measurement, was attached to participants’ right thighs and, thus, recorded only lower extremity movements. As a result, upper extremity activities, such as cooking or doing laundry, were not captured by the sensor, whereas these activites are included in the self-reported PA assessments. These discrepancies likely reflect the well-established divergence between self-reported and sensor-based methods of assessing PA, rather than indicating distinct types of PA. Self-reports are shaped by participants’ perceptions, memory, and interpretation of what constitutes activity, and are often influenced by social desirability or cognitive bias. In contrast, sensor-based data provide an objective account of movement patterns, but lack contextual nuance and may overlook activities perceived as meaningful by individuals. Rather than suggesting one method is inherently more valid, these findings underscore that the two approaches capture different facets of the same behavioral construct. This distinction is particularly important when assessing older adults, where subjective and objective experiences of PA may diverge due to mobility limitations, fall-related concerns, or cognitive factors. Therefore, both assessment types should be interpreted within their methodological contexts, and their combined use may offer a more comprehensive understanding of PA in clinical and research settings (43).  These differences in PA assessment, and in both men and women may explain some of the findings of our study and highlight the importance of considering sex when assessing PA after a fall. Besides sex, future analyses should also consider factors such as the living situation (e.g., living alone or with a relative) (44) and socioeconomic status (45) of an individual, as these may influence PA engagement post-fall, according to previous research.  To evaluate the impact of sensor-based versus questionnaire-based changes in PA on post-fall sequelae, PA assessments should be integrated with direct measurements of functional capacity over an extended observation period. This approach could enable future research to yield deeper insights into the distinct dimensions of PA and their respective utility in understanding post-fall recovery mechanisms in older adults. |
| Limitations | 19 | Discuss limitations of the study, taking into account sources of potential bias or imprecision. Discuss both direction and magnitude of any potential bias | 17-18 | *Limitations*  Given that fewer men than women were recruited in our study, it is important to increase sample sizes in future research, particularly when aiming to identify moderators specific to men. Additionally, extending the follow-up period beyond six months is warranted to gain further insights into the long-term effects and trajectories of PA and related factors in this population. Another limitation of this study was the presence of incomplete datasets, which restricted the statistical analysis and precluded the use of a comprehensive hierarchical model. This was primarily due to the acute aftereffects of the fall and unforeseen challenges during data collection in participants' home environments. Future studies should therefore aim to collect more complete and standardized datasets to enable more robust analytical approaches, including advanced variable selection procedures and multivariable modeling strategies. Moreover, the four-week interval before the first assessment was deliberately chosen to give individuals time to recover sufficiently to participate in a home visit, while still aiming to capture early post-fall behavioral adaptations. Although home visits were ideally scheduled within one week after the ED presentation, logistical and ethical considerations made flexibility necessary. As different time intervals may have influenced PA levels, future studies should consider more frequent and repeated assessments to better account for recovery trajectories and temporal variability in real-world clinical care settings. Although our inclusion criteria were intentionally broad to capture a heterogeneous and realistic population, this diversity may limit the precision of subgroup estimated and generalizability to more narrowly defined populations. Specifically, some participants with cognitive impairment were enrolled with the support of caregivers or family members. Despite this proxy assistance, self‑reported data remain vulnerable to recall and social desirability biases, which may have influenced the accuracy of the reported outcomes. Lastly, the observed change in sensor-based physical activity of 545.8 steps per day six month after a fall lies below reported lower-bound minimal clinically important differences of ~800 steps per day in a study with individuals with multiple sclerosis (46). Given the demographic and clinical differences between populations, the applicability of this threshold is limited. Future studies should aim to define minimal clinically important differences specifical for older adults following a severe fall with ED presentation. |
| Interpretation | 20 | Give a cautious overall interpretation of results considering objectives, limitations, multiplicity of analyses, results from similar studies, and other relevant evidence | 18 | Early identification of moderating factors associated with changes in PA in adults aged 60 years and above may help stratify individuals according to risk and identify those who require further treatment and prevent functional decline. Our findings identified a cut-off age of 66 years and a history of one or more previous falls as significant moderators in females for a reduction of PA. Additionally, unrecovered falls, diminished physical function and concerns about falling should also be considered as early warning signs for both male and female older adults after a severe fall. While our study offers initial insights into moderating factors influencing PA following a severe fall, the exploratory design and limited sample size preclude definitive recommendations for changes in routine ED care. Nonetheless, our findings highlight potential clinical relevance – particularly regarding early identification of high-risk individuals who may benefit from referral to specialized falls clinics for further comprehensive assessments and targeted falls prevention strategies. Future studies with larger populations are needed to validate these results and further explore sex-specific differences in PA trajectories. Feasibility studies should also assess how such approaches could be implemented into clinical workflows and identify barriers and facilitators to integration. |
| Generalisability | 21 | Discuss the generalisability (external validity) of the study results | 18 | Early identification of moderating factors associated with changes in PA in adults aged 60 years and above may help stratify individuals according to risk and identify those who require further treatment and prevent functional decline. Our findings identified a cut-off age of 66 years and a history of one or more previous falls as significant moderators in females for a reduction of PA. Additionally, unrecovered falls, diminished physical function and concerns about falling should also be considered as early warning signs for both male and female older adults after a severe fall. While our study offers initial insights into moderating factors influencing PA following a severe fall, the exploratory design and limited sample size preclude definitive recommendations for changes in routine ED care. Nonetheless, our findings highlight potential clinical relevance – particularly regarding early identification of high-risk individuals who may benefit from referral to specialized falls clinics for further comprehensive assessments and targeted falls prevention strategies. Future studies with larger populations are needed to validate these results and further explore sex-specific differences in PA trajectories. Feasibility studies should also assess how such approaches could be implemented into clinical workflows and identify barriers and facilitators to integration. |
| Other information | |  | | |
| Funding | 22 | Give the source of funding and the role of the funders for the present study and, if applicable, for the original study on which the present article is based | 20 | The project has been funded by the Federal Ministry of Education and Research (BMBF, grant number: 01GY2021). The BMBF did not contribute to designing the study or in writing the manuscript nor has been involved in data collection, analysis and interpretation. |

*Give information separately for cases and controls in case-control studies and, if applicable, for exposed and unexposed groups in cohort and cross-sectional studies.

**Note:** An Explanation and Elaboration article discusses each checklist item and gives methodological background and published examples of transparent reporting. The STROBE checklist is best used in conjunction with this article (freely available on the Web sites of PLoS Medicine at http://www.plosmedicine.org/, Annals of Internal Medicine at http://www.annals.org/, and Epidemiology at http://www.epidem.com/). Information on the STROBE Initiative is available at www.strobe-statement.org.
